# Two Magnetic Orderings and a Spin–Flop Transition in Mixed Valence Compound Mn_3_O(SeO_3_)_3_

**DOI:** 10.3390/ma15165773

**Published:** 2022-08-21

**Authors:** Wanwan Zhang, Meiyan Cui, Jindou Tian, Pengfeng Jiang, Guoyu Qian, Xia Lu

**Affiliations:** 1 School of Materials, Sun Yat-sen University, Guangzhou 510275, China; 2 State Key Laboratory of Structural Chemistry, Fujian Institute of Research on the Structure of Matter, Chinese Academy of Sciences, Fuzhou 350002, China; 3 Department of Chemistry, University of Science and Technology of China, Hefei 230026, China

**Keywords:** mixed-valence, magnetic properties, topological structures

## Abstract

A mixed-valence manganese selenite, Mn_3_O(SeO_3_)_3_, was successfully synthesized using a conventional hydrothermal method. The three-dimensional framework of this compound is composed of an MnO_6_ octahedra and an SeO_3_ trigonal pyramid. The magnetic topological arrangement of manganese ions shows a three-dimensional framework formed by the intersection of octa-kagomé spin sublattices and staircase-kagomé spin sublattices. Susceptibility, magnetization and heat capacity measurements confirm that Mn_3_O(SeO_3_)_3_ exhibits two successive long-range antiferromagnetic orderings with *T*_N1_~4.5 K and *T*_N2_~45 K and a field-induced spin–flop transition at a critical field of 4.5 T at low temperature.

## 1. Introduction

Mixed valence transition metal (TM) oxides with three-dimensional electronic configurations are of great significance in the fields of materials chemistry, electrochemical energy and condensed matter physics due to their diverse crystal structures and electronic configurations [1,2]. From the ancient application of Fe_3_O_4_ in the compass to today’s copper-based high temperature superconducting materials, mixed valence TM oxides exhibit exciting and unusual chemical and physical behaviors, including high-temperature superconductors [3], colossal magnetoresistance [4], ion deintercalation [5], metal-insulator transition [6], electrocatalysis/photocatalysis [7,8], etc. More specifically, copper oxides with bidimensional characters, together with the mixed valency of Cu^+^/Cu^2+^ or Cu^2+^/Cu^3+^, are responsible for superconducting properties [9,10]. The ferromagnetic (FM) material La_0.67_Sr_0.33_MnO_3_ exhibits metallic conductivity due to the Zener double exchange mechanism between Mn^3+^ and Mn^4+^ ions, but BaFe_12_O_19_ (also an FM material) is insulative due to the limitation of the ratio of Fe^2+^ and Fe^3+^ ions [11]. Compound K_2_Cr_8_O_16_ (hollandite), with a rare Cr^3+^/Cr^4+^ mixed valence state, exhibits a metal-insulator transition in a FM state [12]. The transition metal valence state of cathode material LiMO_2_ (M = Mn, Co, Ni) will switch back and forth between M^2+^ and M^4+^ during charging and discharging processes in Li-ion batteries [13,14]. X. Yu et al. reported the experimental observation of skyrmionic bubbles with various topological lattices in colossal magnetoresistive manganite La_1−x_Sr_x_MnO_3_ [15]. In order to discover new materials with unusual physical/chemical properties, it is necessary to explore new mixed-valence transition metal compounds. The compound Mn_3_O(SeO_3_)_3_ (Mn^II^Mn^III^_2_O(SeO_3_)_3_) was first reported by Wildner [16]. Structure analysis confirmed that this compound shows a channel structure with a three-dimensional magnetic topological framework formed by the intersection of octa-kagomé spin sublattices and staircase-kagomé spin sublattices; however, there are few studies regarding its magnetic properties. In this paper, we report the discovery of a mixed valence manganese selenate Mn_3_O(SeO_3_)_3_. Magnetic measurements indicate that this compound possesses two successive antiferromagnetic (AFM) transitions at low-temperature. Moreover, a spin–flop transition is observed at 2 K with an applied magnetic field of ~4.5 T.

## 2. Experimental Section

### 2.1. Synthesis of Mn_3_O(SeO_3_)_3_

**Figure 1:**
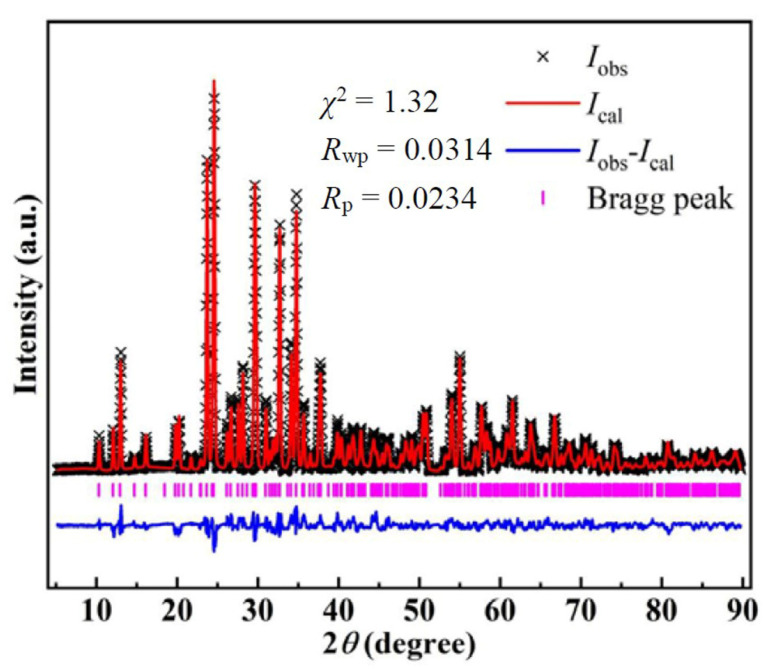
Rietveld refinement of powder X-ray (Cu Kα) diffraction patterns for Mn_3_O(SeO_3_)_3_. The refined lattice constants are *a* = 15.484(9) Å, *b* = 6.665(8) Å, *c* = 9.703(1) Å and *β* = 118.79(4)° with space group *C*2/*m*, which is consistent with the reported parameters of ref. [16].

Single crystals of Mn_3_O(SeO_3_)_3_ were obtained using a conventional hydrothermal method. A mixture of 2 mmol Mn(NO_3_)_2_·xH_2_O (3 N, 0.3943 g), 2 mmol LiI (2 N, 0.2704 g), 1 mmol SeO_2_ (4 N, 0.1110 g) and 5 mL deionized water was sealed in an autoclave equipped with a Teflon liner (28 mL). The autoclave was gradually heated to 230 °C at a rate of 1 °C/min, held for 4 days and then naturally cooled to room temperature. The product contained the desired black noodle-like crystals with a 90% yield. The crystals’ sizes and morphologies were characterized using a stereomicroscope and field emission scanning electron microscopy (FE-SEM, SU8100, Hitachi, Tokyo, Japan). Figure S1 shows images of the crystals under the stereomicroscope and FE-SEM. It can be observed that the crystal size is approximately 0.3 × 0.08 × 0.05 mm. The product’s impurities were manually removed under a microscope. Powdered samples were prepared for physical measurement by crushing small single crystals; purity was confirmed by powder X-ray diffraction (XRD) analysis (Figure 1). Moreover, the reagent LiI acted as a mineralizer, as the quality of crystals was unsatisfactory without it.

### 2.2. Methods

XRD patterns were collected using a Bruker D8 diffractometer with Cu-K_α_ radiation (λ~1.5418 Å) at room temperature. Rieltveld refinement was performed using GSAS-EXPGUI software [17]. Refined crystal structures were analyzed using VESTA software [18]. Furthermore, element analysis was observed using FE-SEM with an X-ray energy-dispersive spectrometer (EDS). EDS analysis confirmed the molar ratio of Mn/Se as 3.1/2.0, which is in good agreement with the X-ray structure analysis. Thermogravimetric analysis (TGA) of Mn_3_O(SeO_3_)_3_ was collected on NETZSCH STA 449C instruments with an Al_2_O_3_ crucible from 50 to 900 °C at a rate of 10 °C/min under N_2_ atmosphere.

Magnetic measurements of a powdered sample of Mn_3_O(SeO_3_)_3_ were performed using a PPMS (Quantum Design, San Diego, CA, USA). The powdered sample (20.6 mg) was placed in a plastic capsule, which was suspended in a copper tube slot. Magnetic susceptibility was measured at 0.1 T from 2 to 300 K. Magnetization was measured at different temperatures at applied field from 0 to 9 T. Heat capacity was measured with the same PPMS system at zero field and determined using a relaxation method on a 5.6 mg sample.

## 3. Results and Discussion

The structure of compound Mn_3_O(SeO_3_)_3_ was first reported by Wildner [16]. Mn_3_O(SeO_3_)_3_ crystallizes in the monoclinic system with the space group *C*2/*m*. As shown in Figure S2, both Mn and Se atoms have three crystallographic sites. The oxidation state is +2 for Mn1 and +3 for Mn2/Mn3. All manganese atoms are coordinated by six oxygen atoms forming MnO_6_ distorted octahedra; Mn–O bond lengths range from 2.100(1) to 2.361(8) Å for Mn1^2+^O_6_ octahedra and from 1.854(2) to 2.310(6) Å for Mn2^3+^O_6_ and Mn3^3+^O_6_, respectively. In other words, the degree of distortion for Mn1^2+^O_6_ octahedra is smaller than that of Mn2^3+^O_6_ and Mn3^3+^O_6_. This is due to the Mn^3+^ (t_2g_^3^e_g_^1^) octahedron with a remarkable Jahn–Teller effect, which may induce a larger structure distortion than Mn^2+^ (t_2g_^3^e_g_^2^). All selenium atoms are in trigonal pyramid geometry with a stereoactive lone pair of 4 s^2^ in Se^4+^ ions; the Se-O bond lengths are approximately 1.70 Å. It should be noted that Se1/Se2/Se3 atoms are surrounded by 4/5/6 manganese atoms with a Se-O-Mn route, respectively. These 4/5/6 manganese atoms contain two Mn^2+^ atoms and 2/3/4 Mn^3+^ atoms, respectively. 

**Figure 2:**
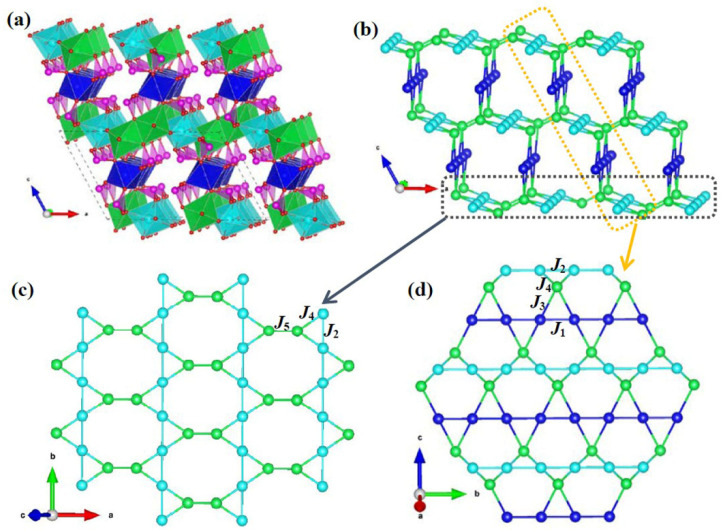
(**a**) The three-dimensional structure frameworks and (**b**) topological spin structures of Mn_3_O(SeO_3_)_3_; (**c**,**d**) show the octa-kagomé and staircase-kagomé spin sublattices, respectively. Here the blue, green, light blue and pink polyhedra represent Mn1O_6_, Mn2O_6_, Mn3O_6_ and SeO_3_, respectively. Balls of the above colors represent Mn1, Mn2 and Mn3 ions, respectively.

**Figure 3:**
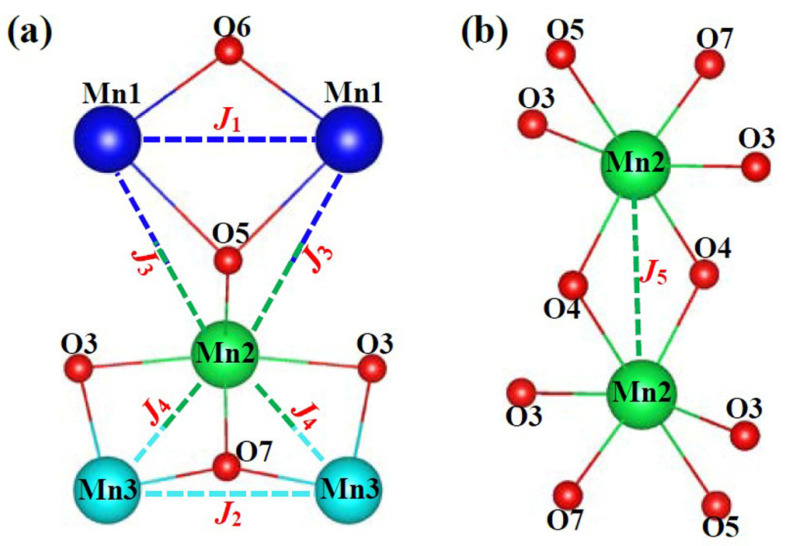
Detailed linkages of five main superexchange pathways in Mn_3_O(SeO_3_)_3_. (**a**) indicates *J*_1_ to *J*_4_ and (**b**) indicates *J*_5_, respectively.

As shown in Figure 2, Mn_3_O(SeO_3_)_3_ shows a tunnel structure along the b-axis, in which the framework is constituted by MnO_6_ octahedra and SeO_3_ trigonal pyramids. Mn1O_6_ octahedra share their edges (O5–O6) to form uniform [-Mn1-] chains along the b-axis. Mn^3+^O_6_ octahedra are interconnected via edge-sharing oxygen atoms, forming a two-dimensional [-Mn^3+^-] layered structure parallel to (001). The detailed linkage mode between manganese ions is shown in Figure 3. Two Mn2O_6_ octahedra connect to each other by edge-sharing oxygen atoms (O4–O4) to form a [Mn_2_O_10_] dimer along the a-axis. The Mn2-O4-Mn2 angle is 101.34(9)°. Mn3O_6_ octahedra are interconnected by corner-sharing oxygen atoms (O7) to form uniform [-Mn3-] chains along the b-axis. One Mn2O_6_ octahedron and two Mn3O_6_ octahedra are connected in an isosceles triangle configuration. The neighbored [-Mn^3+^-] layers are separated by [-Mn1-] chains and SeO_3_ trigonal pyramids. Furthermore, we noted that Mn1O_6_ octahedra are interconnected with Mn2O_6_ octahedra via conner-sharing O5 atoms, but Mn1O_6_ and Mn3O_6_ octahedra are connected by SeO_3_ groups in the manner of Mn1-O-Se-O-Mn3. After removing the nonmagnetic SeO_3_^2−^ groups, the topological arrangement of magnetic Mn ions is a three-dimensional framework (Figure 2b). Mn^3+^ ions form a two-dimensional octa-kagomé lattice parallel to (001) (Figure 2c). The adjacent octa-kagomé layers are connected by [-Mn1-] chains. It is significant that there is a staircase-kagomé lattice composed of Mn^2+^ and Mn^3+^ parallel to (100) in the magnetic topological framework (Figure 2d). The shortest Mn–Mn distance in both [-Mn1-] and [-Mn3-] chains is 3.332(9) Å. However, the detailed connection mode of MnO_6_ octahedra in [-Mn1-] chains are edge-sharing, whereas in [-Mn3-] chains it is corner-sharing. The Mn-O-Mn angles in [-Mn1-] and [-Mn3-] chains are 89.77(1)°/102.90(8)° and 127.61(5)°, respectively. The Mn2--Mn2 and Mn2–Mn3 distances in the octa-kagomé lattice are 3.150(0) Å and 3.069(6) Å, respectively, whereas the Mn1–Mn2 distance is 3.902(1) Å.

**Figure 4:**
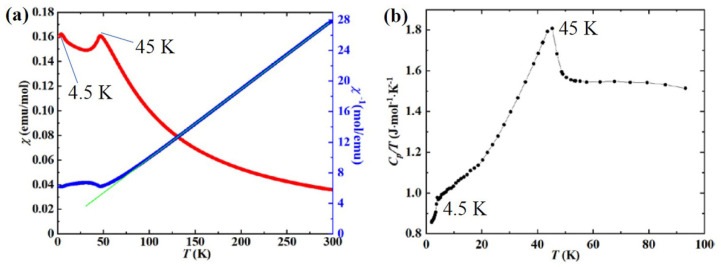
(**a**) The magnetic susceptibility *χ*(*T*) of Mn_3_O(SeO_3_)_3_ and its reciprocal. (**b**) The heat capacity of Mn_3_O(SeO_3_)_3_. The green solid line indicates Curie–Weiss fitting.

Figure 4a shows the temperature dependence of magnetic susceptibility *χ*(*T*) of Mn_3_O(SeO_3_)_3_ measured at 0.1 T. Magnetic susceptibility increases with decreasing temperature; two peaks can be observed at *T*_N1_~4.5 K and *T*_N2_~45 K., showing AFM transitions. At high temperature (80–300 K) inverse susceptibility *χ*^−1^(*T*) follows the Curie–Weiss law with a Weiss temperature of *θ* = −8.89 K and a Curie constant of *C* = 11.03 emu·mol^−1^·K. The effective magnetic moment is calculated to be *μ*_eff_ = 5.42(3) *μ*_B_, obtained by *μ*^2^_eff_ = 8*C/n*, where *n* = 3. This value of *μ*_eff_ is slightly smaller than the spin-only value of 5.91(6) *μ*_B_ for Mn^2+^ (3d^5^, high spin) and larger than the spin-only value of 4.89(9) *μ*_B_ for Mn^3+^ (3d^4^, high spin). As Mn ions are mixed-valent, the theoretical magnetic moment of the titled compound is *μ*_theo_ = 5.25(9) *μ*_B_ obtained by the equation *μ*^2^_eff_ = [*μ*^2^_eff_ (Mn^2+^) + 2*μ*^2^_eff_ (Mn^3+^)]/3. The value of *μ*_eff_ is quite close to that of *μ*_theo_, confirming that Mn ions in the structure are mixed valence. The negative value of *θ* suggests the presence of dominative AFM interactions between neighboring Mn ions. Figure S3 shows the *χT*-*T* curve, in which the value of *χT* decreases with decreasing temperature, which is characteristic of typical AFM interactions. As shown in Figure 4b, the heat capacity data of Mn_3_O(SeO_3_)_3_ show a λ-type peak at T~45 K and a corner-type transition at 4.5 K, providing concrete evidence for the two long-range magnetic orderings observed in the magnetic susceptibility curves.

**Figure 5:**
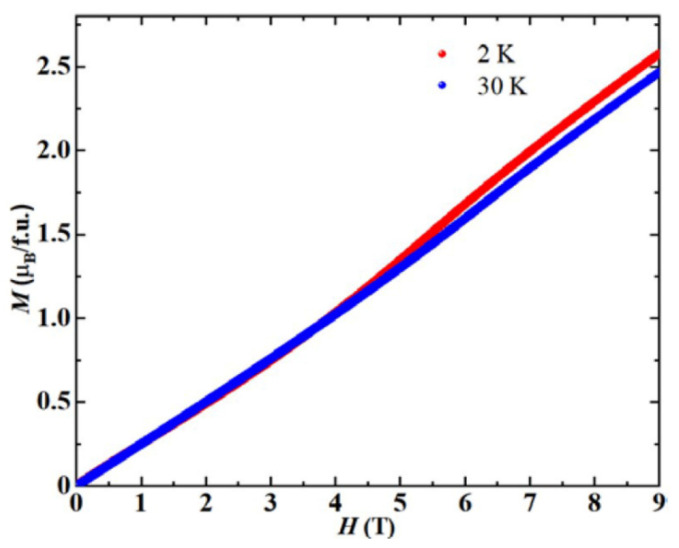
Isothermal magnetization (*M*) as a function of applied field (*H*) at 2 K and 30 K.

To further investigate the magnetic properties of the system, magnetization (*M*) as a function of applied field (*H*) was observed at 30 K and 2 K. As shown in Figure 5, at 30 K, magnetization increased linearly with increasing field, and did not saturate at 9 T. Furthermore, no hysteresis or remanent magnetization was observed. These features of the *M*–*H* curve suggest that the magnetic anomaly at *T*~45 K is the onset of an AFM ordering. At 2 K, the magnetization (*M*) shows a linear increase in magnetization at low field, indicative of a characteristic AFM ground state. A clear change in slope in the magnetization is observed at approximately 4.5 T, indicating field-induced magnetic transition. Furthermore, no hysteresis can be observed on the *M*–*H* curve.

It is well-known that the magnetic properties of solid magnets are strongly related to their structural features. The three-dimensional manganese topological framework of Mn_3_O(SeO_3_)_3_ is formed by the intersection of octa-kagomé lattices and staircase-kagomé lattices. Firstly, we know that the kagomé-like lattices containing equilateral or isosceles triangle sublattices may exhibit strong frustrated magnetic properties [19,20,21]. We note the value of frustration factor, *f* =|*θ*|/*T*_N_~0.20, with Weiss temperature *θ* = −8.89 K and Neel temperature *T*_N2_~45 K, ruling out spin frustration in the system. It is well known that primary magnetic interactions originate from the superexchange of Mn-O-Mn. A detailed description of superexchange interactions is shown in Figure 2c,d and itemized in Table 1; there are five main magnetic exchange interactions, numbered *J*_1_–*J*_5_, within the three-dimensional spin-lattice according to the Goodenough–Kanamori–Anderson rules (GKA rules) [22]. There are two isosceles triangular topological configurations composed by *J*_1_–*J*_4_ on the staircase-kagomé spin sublattice. According to the GKA rules, *J*_2_ and *J*_3_ are AFM interactions. *J*_1_ and *J*_4_ both have two Mn-O-Mn superexchange interactions, as the corresponding MnO_6_ octahedra share their edges. In general, the spin exchange parameter, *J*, can be written as *J = J*_FM_ *+ J*_AFM_, where *J*_FM_ indicates ferromagnetic exchange and *J*_AFM_ indicates antiferromagnetic exchange (*J*_FM_ > 0 and *J*_AFM_ < 0) [23]. So, *J*_1_ = *J*_1FM(O5)_ *+ J*_1AFM(O6)_, and the AFM interaction via O6 in *J*_1_ is negligible; this means that *J*_1_ ≈ *J*_1FM(O5)_ > 0. Using the same analytical method, the spin exchange parameter, *J*_4_, is ambiguous, as the magnitude of *J*_4AFM(O7)_ is difficult to calculate. *J*_5_ should be a weak AFM interaction. This analysis indicates that AFM interactions are dominant in the system, which is consistent with the negative value of *θ*. It is significant that the neighboring octa-kagomé sublattices are separated by [-Mn1-] chains and that the neighboring staircase-kagomé sublattices are connected by [Mn_2_O_10_] dimers. It is safely said that these two long-range magnetic orders are driven by interlayer magnetic coupling. It is noteworthy that [-Mn1-] chains are composed of Mn^2+^ ions. If Mn^2+^ ions are replaced with nonmagnetic ions with a radius similar to Mn^2+^, such as Mg^2+^ or Zn^2+^ [24], an octa-kagomé lattice composed of Mn^3+^ ions might form. The expected compounds may exhibit spin-liquid or other quantum physical properties [25,26]. Exploratory synthesis of related compounds is underway.

**Figure 6:**
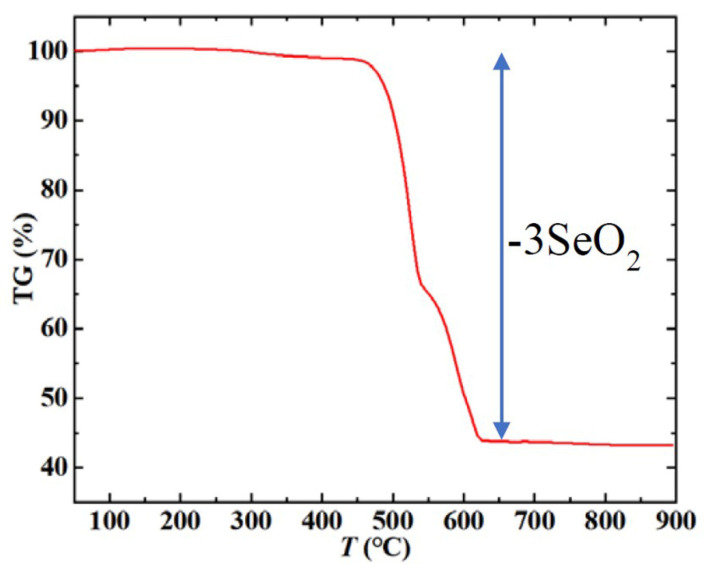
The thermogravimetric curve of Mn_3_O(SeO_3_)_3_, which shows that the residual product of decomposition above 630 °C is Mn_3_O_4_.

As shown in Figure 6, Mn_3_O(SeO_3_)_3_ undergoes a slow weight gain of approximately 0.20% from 100 to 200 °C and a slow weight loss of approximately 0.40% from 300 to 400 °C. As the weight of the sample is only approximately 7 mg, slight weight gain/loss may be caused by instrument error. As the temperature rises, two successive steps of weight loss of approximately 57% occur from 450 to 630 °C, corresponding to the calculated 59% loss of 3SeO_2_. The 2% difference may also be caused by instrument error. Based on this analysis, the final residues of Mn_3_O(SeO_3_)_3_ should be Mn_3_O_4_; however, this was difficult to characterize, as the residues melted in the Al_2_O_3_ crucible after being heated to 900 °C. We re-selected the sample and sintered it in a smooth quartz crucible at 800 °C in a nitrogen atmosphere for 10 min. We then scraped off the sintered product and performed powder X-ray diffraction analysis. As shown in Figure S4, the residue was confirmed as Mn_3_O_4_ (PDF #80-0382). This result is consistent with the decomposition characteristics of most manganese-based compounds.

## 4. Conclusions

A mixed-valence compound, Mn_3_O(SeO_3_)_3_, was successfully synthesized using a conventional hydrothermal method. The reagent LiI acted as a mineralizer. Mn_3_O(SeO_3_)_3_ was shown to have a channel structure with a three-dimensional magnetic topological framework formed by the intersection of octa-kagomé spin sublattices and staircase-kagomé spin sublattices. Magnetic and specific heat data confirmed that Mn_3_O(SeO_3_)_3_ exhibits two successive long-range AFM orderings with *T*_N1_~4.5 K and *T*_N2_~45 K, and a field-induced spin–flop at a 4.5 T critical field at low temperature. Moreover, magnetic measurements confirmed that the ratio of Mn^2+^/Mn^3+^ ions in this compound is 1:2, which is consistent with the structural analysis. The exploratory synthesis of Mg^2+^ or Zn^2+^ replaced compounds is in progress.

## Figures and Tables

**Table 1:** Geometric parameters of dominant magnetic superexchanges in Mn_3_O(SeO_3_)_3_ *.

*J*_1_	d(Mn-O) (Å)	d(Mn-Mn) (Å)	Mn-O-Mn Angle (°)	Magnetism
*J*_1_	Mn1-2.130(7)-O6-2.130(7)-Mn1 Mn1-2.361(4)-O5-2.361(4)-Mn1	3.332(9)	Mn1-O6-Mn1…102.90(8)_AFM-w_ Mn1-O5-Mn1…89.77(1)_FM-S_	FM
*J*_2_	Mn3-1.857(1)-O7-1.857(1)-Mn3	3.332(9)	Mn3-O7-Mn3…127.61(5)_AFM-S_	AFM
*J*_3_	Mn1-2.361(4)-O5-2.278(1)-Mn2	3.902(1)	Mn1-O5-Mn2…114.49(4)_AFM-S_	AFM
*J*_4_	Mn2-2.049(5)-O3-2.310(4)-Mn3 Mn2-1.854(2)-O7-1.857(1)-Mn3	3.069(6)	Mn2-O3-Mn3…89.29(7)_FM-S_ Mn2-O7-Mn3…111.59(8)_AFM-S_	?
*J*_5_	Mn2-1.896(5)-O4-2.169(5)-Mn2 Mn2-2.169(5)-O4-1.896(5)-Mn2	3.150(0)	Mn2-O4-Mn2…101.34(9)_AFM-w_ × 2	AFM

* The S or W behind AFM or FM (in the Mn-O-Mn Angle (°) column) refers to the magnitude of the *J* value; S refers to strong and W refers to weak.

## Data Availability

Data is contained within the article or supplementary material.

## Supplementary Materials

The following supporting information can be downloaded at: https://www.mdpi.com/article/10.3390/ma15165773/s1, Figure S1. Single crystals of Mn_3_O(SeO_3_)_3_ obtained by a conventional hydrothermal method. Figure S2. The oxygen-coordination environments for (a) Mn1, (b) Mn2, (c) Mn3, (d) Se1, (e) Se2 and (f) Se3 atoms in Mn_3_O(SeO_3_)_3_. Figure S3. The variation in *χT* with the temperature of Mn_3_O(SeO_3_)_3_. With the decreasing temperature, the value of *χT* decreases. Figure S4. Powder X-ray diffraction pattern for the final residues of Mn_3_O(SeO_3_)_3_ after sintered at 800 °C for 10 min in nitrogen atmosphere.
