# Supplementary material for: Two Magnetic Orderings and a Spin–Flop Transition in Mixed Valence Compound Mn3O(SeO3)3
Source: Materials (Basel). 2022 Aug 21;15(16):5773. doi: 10.3390/ma15165773 (PMC9414948; doi:10.3390/ma15165773)
Supplement: Supplementary file 1 [file materials-15-05773-s001.zip › materials-1830401-supplementary.pdf]

Two Magnetic orderings and a spin-flop transition in mixed valence compound $Mn_3O(SeO_3)_3$

Wanwan Zhang [1], Meiyan Cui [2], Jindou Tian [3], Pengfeng Jiang [1], Guoyu Qian [1, *], and Xia Lu [1]

[1] School of Materials, Sun Yat-sen University, Guangzhou 510275, P. R. China.

[2] State Key Laboratory of Structural Chemistry, Fujian Institute of Research on the Structure of Matter, Chinese Academy of Sciences, Fuzhou, Fujian 350002, China.

[3] Department of Chemistry, University of Science and Technology of China, Hefei, Anhui 230026, China.

E-mail: qiangy@mail.sysu.edu.cn

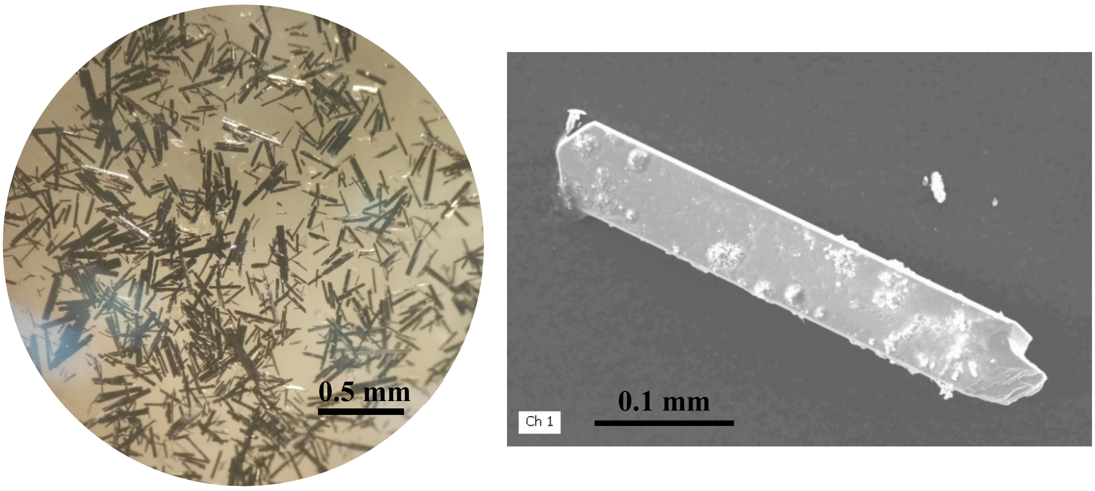

(a) **(b)**

Figure S1. Single crystals of $Mn_3O(SeO_3)_3$ obtained by a conventional hydrothermal method. (a) and (b) are the photos under optical microscope and scanning electron microscopy, respectively.

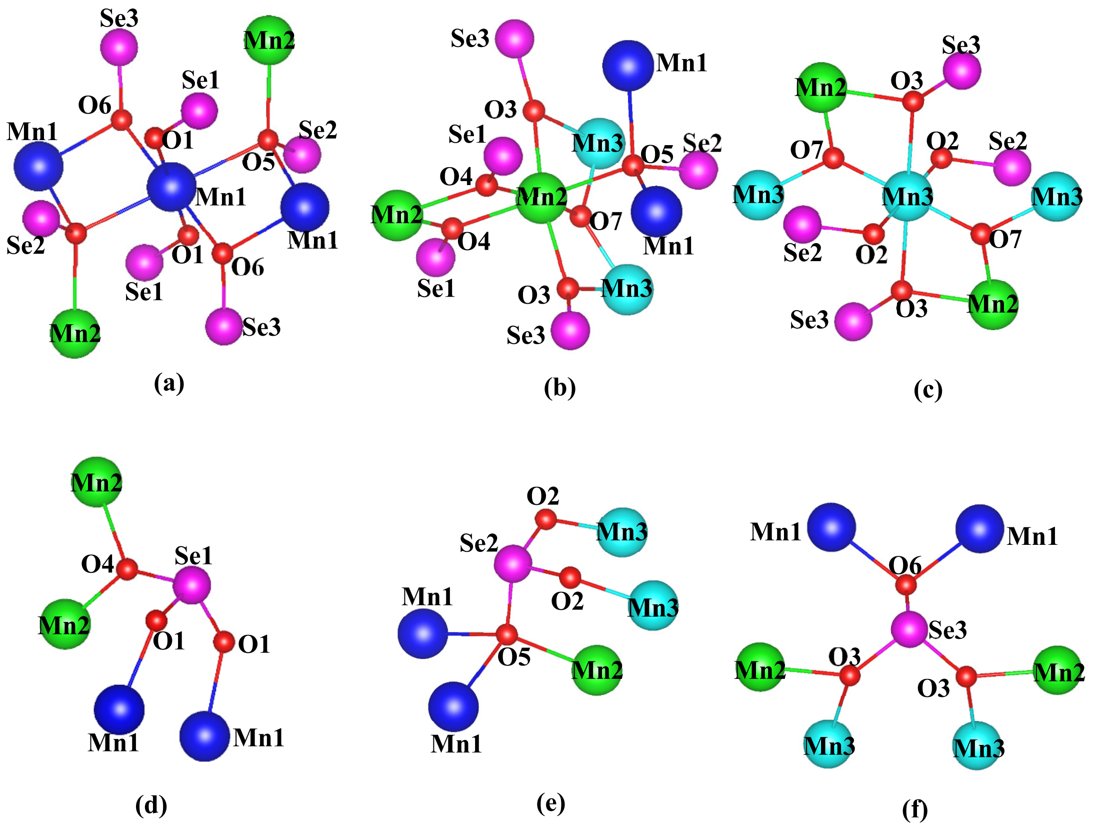

Figure S2. The oxygen-coordination environments for (a) Mn1, (b) Mn2, (c) Mn3, (d) Se1, (e) Se2 and (f) Se3 atoms in $Mn_3O(SeO_3)_3$.

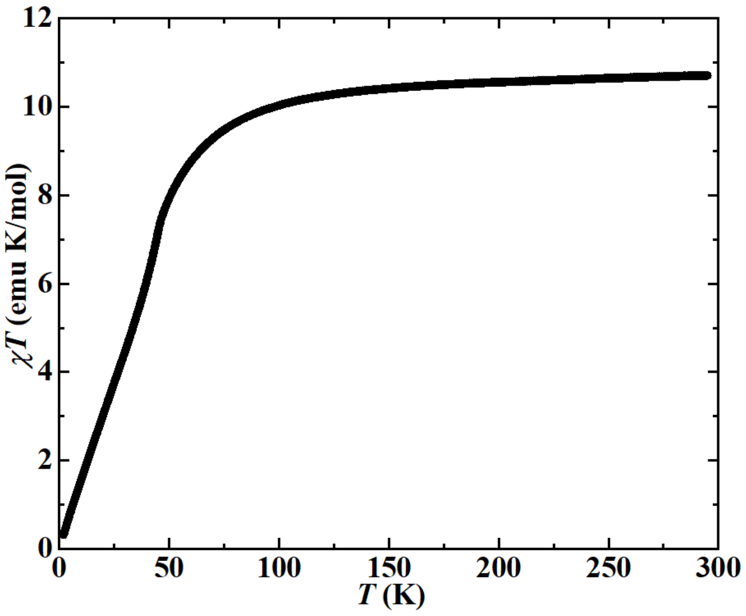

Figure S3. The variation of χT with the temperature of $Mn_3O(SeO_3)_3$. With the decreasing temperature, the value of χT decreases.

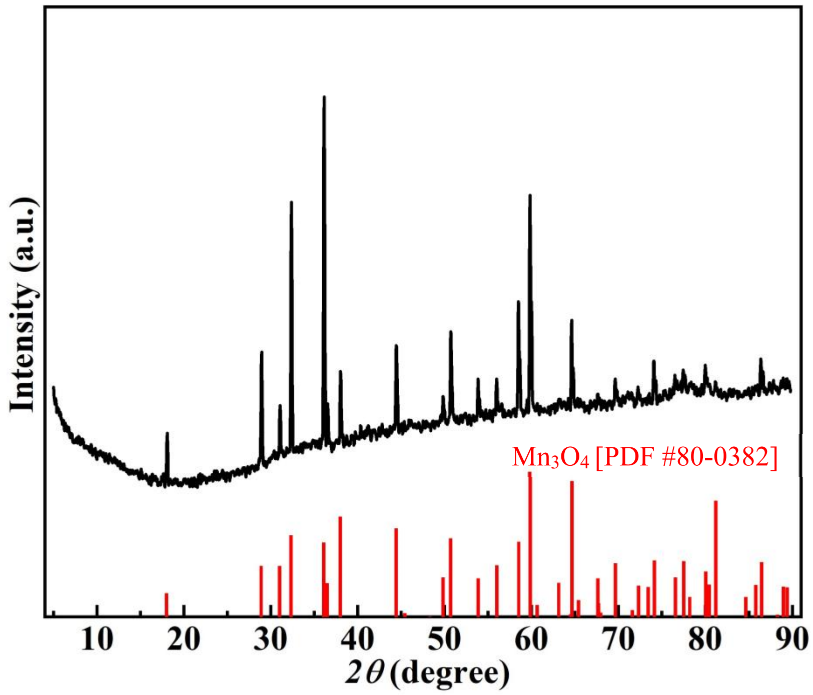

Figure S4. Powder X-ray diffraction pattern for the final residues of $Mn_3O(SeO_3)_3$ after sintered at 800 ℃ for 10 minutes in nitrogen atmosphere.
